# Role of the Conjoined Tendon in Hip Stability Post-Total Hip Arthroplasty: Insights From a Direct Anterior Approach Cadaver Study

**DOI:** 10.1016/j.artd.2024.101487

**Published:** 2024-10-15

**Authors:** Gongyin Zhao, Chenyu Zhao, Hongwei Bao, Junting Liu, Baojun Zhou, Yuji Wang

**Affiliations:** a Department of Orthopedics, The Affiliated Changzhou Second People’s Hospital of Nanjing Medical University, Changzhou Medical Center, Nanjing Medical University, Changzhou, China; b Department of Orthopedic Biomechanics Research Laboratory, Mayo Clinic, Rochester, MN, USA; c Dalian Medical University, Dalian, China; d Department of Orthopedics, Jingjiang People's Hospital, Taizhou, China; e Department of Acute Care Surgery, The First Affiliated Hospital of Guangxi Medical University, Nanning, China; f Department of Orthopedics, The Third Affiliated Hospital of Gansu University of Chinese Medicine, Baiyin, China; g Department of Orthopedic Surgery and Biochemistry & Molecular Biology, Mayo Clinic, Rochester, MN, USA

**Keywords:** Direct anterior approach, Total hip arthroplasty, Conjoined tendon, Piriformis tendons, Dislocation

## Abstract

**Background:**

Hip dislocation represents a significant complication encountered following hip arthroplasty procedures. In this investigation, we conducted a comparative analysis of the biomechanical characteristics exhibited by the piriformis and the conjoined tendon after total hip arthroplasty (THA) via the direct anterior approach (DAA), utilizing cadaveric specimens. The objective is to ascertain the relative significance of the piriformis muscle and the conjoined tendon in mitigating hip dislocation.

**Methods:**

A total of 16 hip joints from 8 freshly frozen cadavers were selected and stratified into 2 groups: the piriformis tendon (PT) group and the conjoined tendon (CT) group. Following THA via the DAA, measurements were taken to record the torque required to induce hip dislocation under various conditions. Torque readings were obtained with the tendon in its intact state (intact group) and after preservation or reconstruction of either the PT or the CT.

**Results:**

The torques of anterior and posterior dislocation in PT group were 22.24 ± 4.53 N.m and 30.7 ± 15.5 N.m following tendon severed, and 20.04 ± 2.67 N.m and 17.5 ± 6.7 N.m following reconstruction. There were no differences compared to the intact group. The torque decreased in the CT group after CT was severed with the torques dropping from 31.2 ± 7.6 N.m to 8.18 ± 2.6 N.m (*P* < .0001) for anterior dislocation, and from 34.9 ± 8.3 N.m to 9.8 ± 2.8 N.m (*P* < .0001) for posterior dislocation. Following reconstruction, the torque required for dislocation significantly increased (*P* < .0001).

**Conclusions:**

This study underscores the preeminent role of the CT in ensuring hip stability following THA via DAA, highlighting the cruciality of its preservation and reconstruction during surgical interventions.

## Introduction

The stability of the joint after total hip arthroplasty (THA) is influenced by several factors, including prosthetic positioning, static stabilizers (ligaments and capsules), and dynamic stabilizers (muscles and tendons) across the hip joint. Despite advancements in prosthesis design and surgical techniques, hip dislocation remains a concern due to the increasing number of THA procedures performed. [[1], [2], [3], [4]] The soft-tissue damage or imbalance surrounding the hip joint is considered a contributing factor to hip dislocation. [[5], [6], [7], [8]] To address this concern, the direct anterior approach (DAA) has become increasingly favored among hip joint surgeons. This approach utilizes the interval between the sartorius and tensor fasciae latae muscles. [[9], [10], [11], [12]] However, proficiency in this technique requires rigorous training, especially in the precise identification and management of anatomical structures such as the short external rotator muscle and the partial posterior joint capsule. [[13], [14], [15]]

THA performed via the DAA often necessitates the release of soft tissue such as the piriformis, tensor fasciae latae, conjoined tendon (CT), and iliopsoas muscles to achieve sufficient exposure. [16,17] Among DAA surgeons, the preferred option for managing challenging cases is releasing the PT and CT, as opposed to releasing the tensor fasciae latae and iliopsoas muscles, which may negatively impact postoperative function. [18]

Studies indicate that intraoperative damage to crucial stabilizing structures, including the obturator externus, PT, and CT, can contribute to hip dislocation. [6,[19], [20], [21]] The piriformis tendon (PT) has garnered attention due to its origins in the anterior sacrum and sacroiliac joint, its passage through the greater sciatic foramen via the sciatic notch, and its insertion into the greater trochanter (GT). [22,23] Research underscores the critical role of the PT in maintaining hip stability. [23,24] Additionally, the CT, the largest among the short external rotators, is believed to have the most substantial influence on hip stability. [17,25,26] Its role in stabilizing the hip joint is considered crucial, highlighting the importance of preserving this structure during THA procedures.

Recent research has focused on the contribution of the posterior capsule, which includes the iliofemoral, pubofemoral, and ischiofemoral ligaments to hip stability. [14,[27], [28], [29]] It is noteworthy that the PT and CT are more easily identifiable and of particular interest compared to the ligaments integrated into the joint capsule. Therefore, further investigation is warranted to understand the mechanisms underlying dislocation associated with the PT and CT tendons better and to enhance our understanding of this complex issue.

The objective of this cadaveric study is to identify structures that exhibit greater resistance to anterior-posterior dislocation by measuring and comparing the torsional forces required for dislocation. Through this investigation, we aim to ascertain the relative importance of the PT and CT tendons in maintaining hip stability.

We hypothesize that the CT plays a significant role in restricting hip dislocation. This research endeavor aims to quantify the torque necessary for posterior dislocation of the hip joint with and without these tendons and comparing the significance of the CT and the PT in preserving hip stability.

## Material and methods

### Subjects

The study protocol was approved by the institutional review board to ensure ethical standards and patient confidentiality. A total of 8 freshly frozen cadavers, comprising 4 males and 4 females, were included in this study, resulting in a total of 16 hips examined. The exclusion criteria were: 1) documented hip trauma or surgical history; 2) body mass index over 30; and 3) freezing duration exceeding 6 months. The mean age at death for the cadaver donors was 73.9 years, with a range of 59-86 years. Detailed information about the individual donors is provided in Table 1.

**Table 1:** Baseline characteristics of the donor population and postoperative outcomes.

Variable	PT	CT	*P*-value
Age (years)	75.25 ± 5.68	75.75 ± 3.31	.7608
BMI (kg/m^2^)	20.13 ± 1.74	21.6 ± 1.96	.4164
Gender, male/female	2/2	2/2	N/A
Anteversion angle (°)	19.93 ± 2.15	17.8 ± 2.34	.0931
Inclination angle (°)	42.33 ± 1.31	41.05 ± 1.99	.0697
Length difference (mm)	0.5 ± 1.93	0.25 ± 1.99	.5796

*P*-values determined using paired *t*-test.

BMI, body mass index; anteversion angle, anteversion angle of cup; inclination angle, inclination angle of cup; length difference, preoperative and postoperative length difference.

### Procedure

The procedure was performed by a surgeon (Y.J.W.) and his team, who have experience with over 1000 THA procedures via DAA. The cadaveric specimens were positioned supine, and the surgical procedure was performed using DAA. Specifically, the surgical procedure involved anterior access to the hip through the tensor fasciae latae, with an osteotomy performed approximately 1.5 cm above the lesser trochanter. The acetabulum was positioned at an approximate angle of 45° of external inclination and 20° of anteversion. Intraoperative fluoroscopy was utilized and adjusted to ensure uniform position of the cup across all specimens.

The posterior capsule was meticulously dissected to optimize exposure of the PT and CT. Proximal femoral elevation was achieved using a hook and retractor, with adjustments made as necessary to lower the distal surgical table for better exposure. The surgical procedures were performed with utmost caution to safeguard the integrity of the PT and CT, minimizing disruption or damage to these vital structures. The positioning of the stem was determined by the surgeon based on his experience. The 16 hips were categorized into 2 distinct groups: the PT group and the CT group. In the PT group, the PT was selectively cut at the point where it intersected into the GT, while preserving the integrity of the CT. Conversely, in the CT group, the CT was cut at the point where it intersected with the GT, while preserving the PT. Considering the study's cost and the desired outcomes, a short-stem (SMF, Smith & Nephew, USA) was employed as the femoral component, while the acetabular component consisted of a cup system and ceramic liner without an augmented rim (CombiCup, Link, Germany). In cases of loosened acetabulum, 2 25 mm screws were used for reinforcement.

After the arthroplasty procedure, the surgical side's knee was exposed to facilitate the retrograde insertion of a femoral nail (10 mm × 36 mm, Wego, China). This nail was carefully positioned from the femoral condyle extending to the distal end of the prosthesis, and a locking nail was implanted at the distal end to prevent rotation. Subsequently, a torque-measuring apparatus was affixed to the end of the intramedullary nail to facilitate the application of torque. [30,31] In the postoperative phase, the anterior pelvic tilt angle was quantified indirectly using radiographic imaging, following the methodology described by Liaw et al. [32]

**Figure 1:**
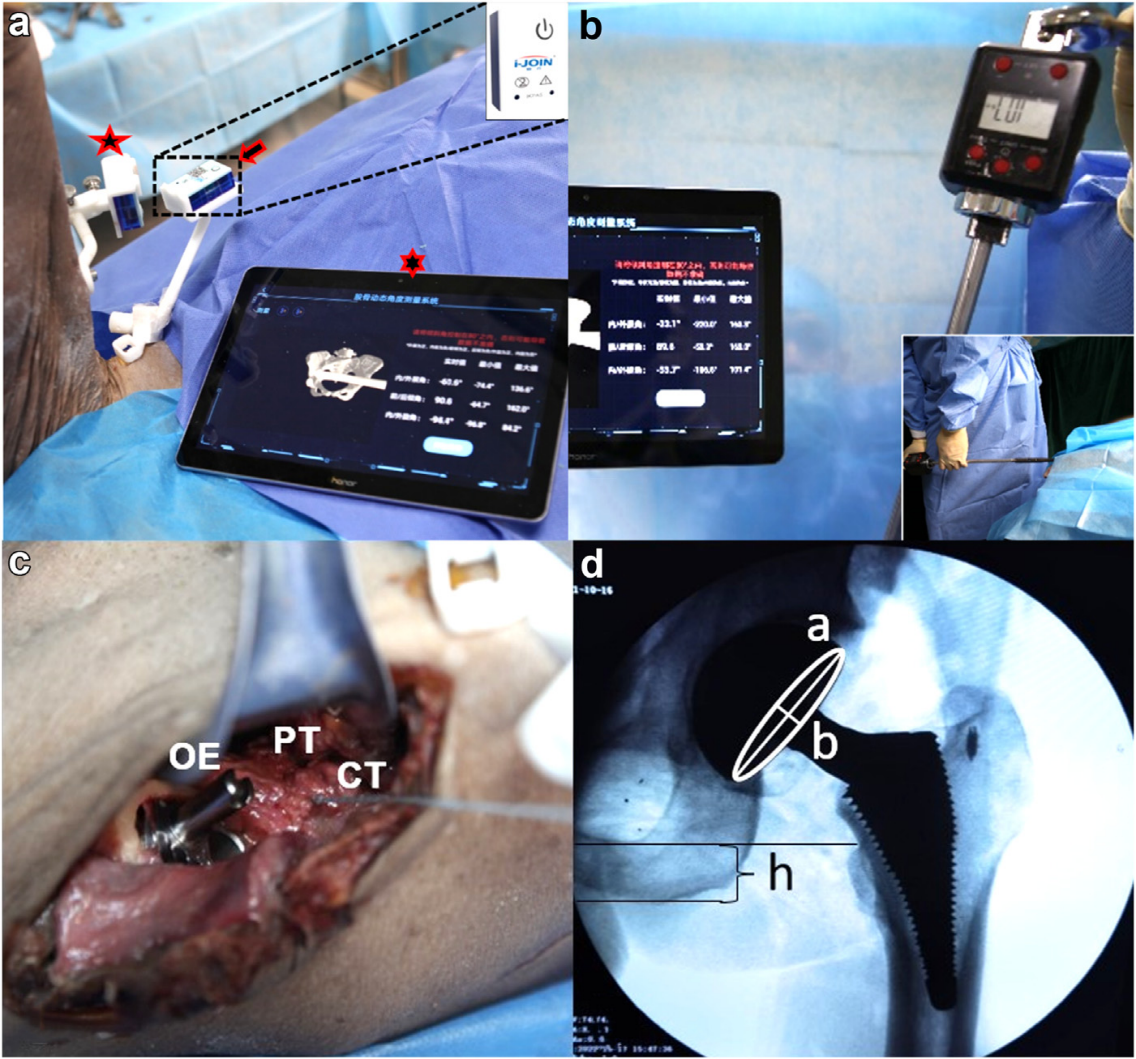
(a) I-joint Mini Navigation System. The pentagon is the measurement module, the arrow points to the reference module, and the hexagon is the monitor. (b) Torque testing for hip dislocation. (c) Anchor repair of the joint tendon. (d) Intraoperative X-ray fluoroscopic evaluation. a: Long axis of the acetabular projection; b: short axis of the acetabular projection; h: distance from the lesser trochanter to the sciatic bone.

During the procedure, we utilized a navigation system (I-joint Mini Navigation System, Shanghai, China). The positioning module was securely attached to the anterior superior iliac spine, while the measurement module was positioned at the midpoint of the femur, specifically at the distal end of the prosthesis (Fig. 1a). The hip joint was maintained in flexion at approximately 100° and a slight adduction of 10°-15°. To induce dislocation, internal rotation torque was applied to the femur by performing a clockwise (right) or counterclockwise (left) twisting motion on the torsion adapter (Fanyaa, China) connected to the end of the intramedullary nail. This rotation continued until dislocation occurred. For the evaluation of anterior dislocation, the lower extremity was gently extended by 15° and adducted by 30°. The peg was then gradually rotated in a counterclockwise (right) or clockwise (left) direction until anterior dislocation was observed. (Fig. 1b)

**Supplementary Figure 1:**
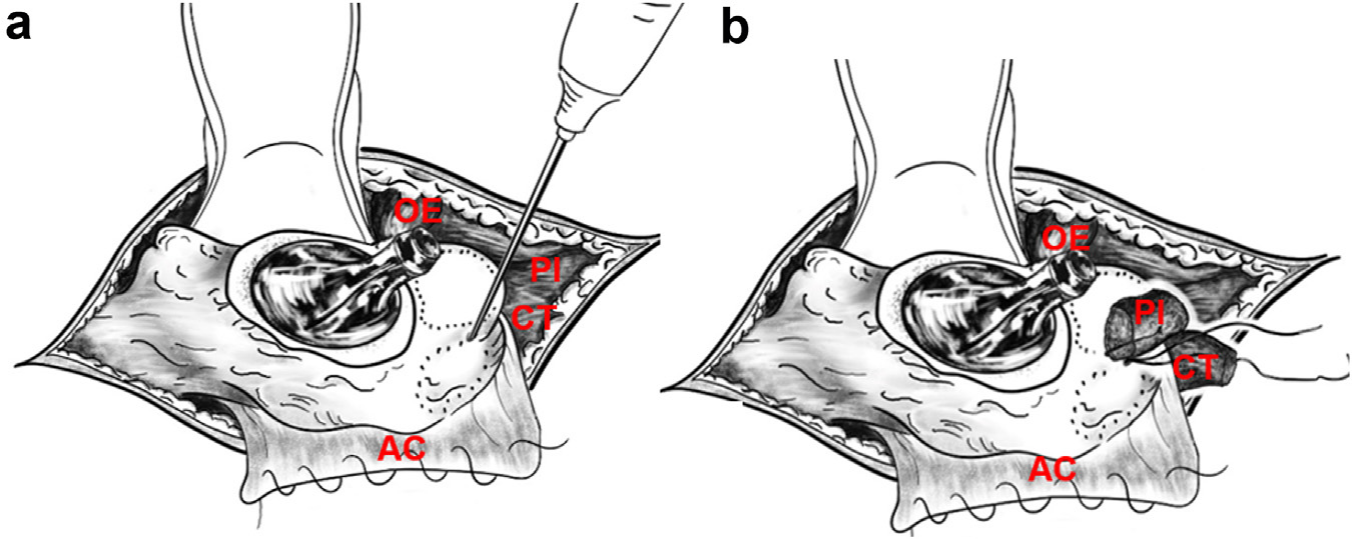
(a) Before and (b) after reconstruction of the piriformis muscle. OE, obturator externus tendon; PI, piriformis tendon; CT, conjoined tendon; AC, anterior capsule.

During the reconstruction phase following the torque measurements, the hip joint was deliberately dislocated. A 3.5 mm suture anchor (Smith & Nephew, USA) was securely attached to the anatomic footprint of piriformis and cojoined tendon for stabilization and reinforcement. The PT or CT was meticulously sutured to the GT using appropriate sutures. (Fig.1c, Supplementary Fig. 1)

To assess the length of the lower extremity, we utilized a standard intraoperative fluoroscopic pelvic anteroposterior view to measure the position of the lesser trochanter. This involved calculating the difference in distance between the preoperative and postoperative measurements from the lesser trochanter to the sciatic bone. Simultaneously, we adjusted the size of heads and stems according to the intraoperative fluoroscopic to ensure consistency of the offset. Additionally, we determined the anteversion angle by measuring the length of the acetabular cup in both the long and short axes. [33] (Fig. 1d) Torque measurements for anterior and posterior dislocations were conducted at 3 distinct time points for each hip joint: prior to any tendon intervention, after tendon severance, and following tendon reconstruction.

### Statistical analysis

In our statistical design, we established the effect size (Cohen's f) at 0.8, with an alpha error probability (type I error rate) set at 0.05 and statistical power (1 - β) at 0.8. Utilizing these parameters, we calculated the required sample size using G∗Power 3.1 software. The outcome indicated a total sample size of 21 subjects, equating to at least 7 samples per group. One-way analysis of variance was used for statistical analysis. *P*-value <.05 was considered statistically significant. Data were analyzed by GraphPad Prism 5 software (GraphPad Software, Inc., USA).

## Results

An exceptional case was noted within the piriformis group consisting of 8 specimens, where one specimen exhibited significantly heightened resistance to anterior dislocation. Specifically, this specimen necessitated 56.8 N.m of torque for anterior dislocation with all tendons intact and 55.5 N.m of torque when the piriformis muscle was severed. A Shapiro-Wilk normality test revealed a *P*-value of .004 for the piriformis group during anterior dislocation, indicating a departure from normal distribution. After excluding this outlier, the remaining dataset met the criteria for normal distribution.

The anteversion angle of the specimens in both groups was measured with no significant difference observed. Additionally, there was no significant difference in the length of the lower limbs between the groups. (Table 1)

**Figure 2:**
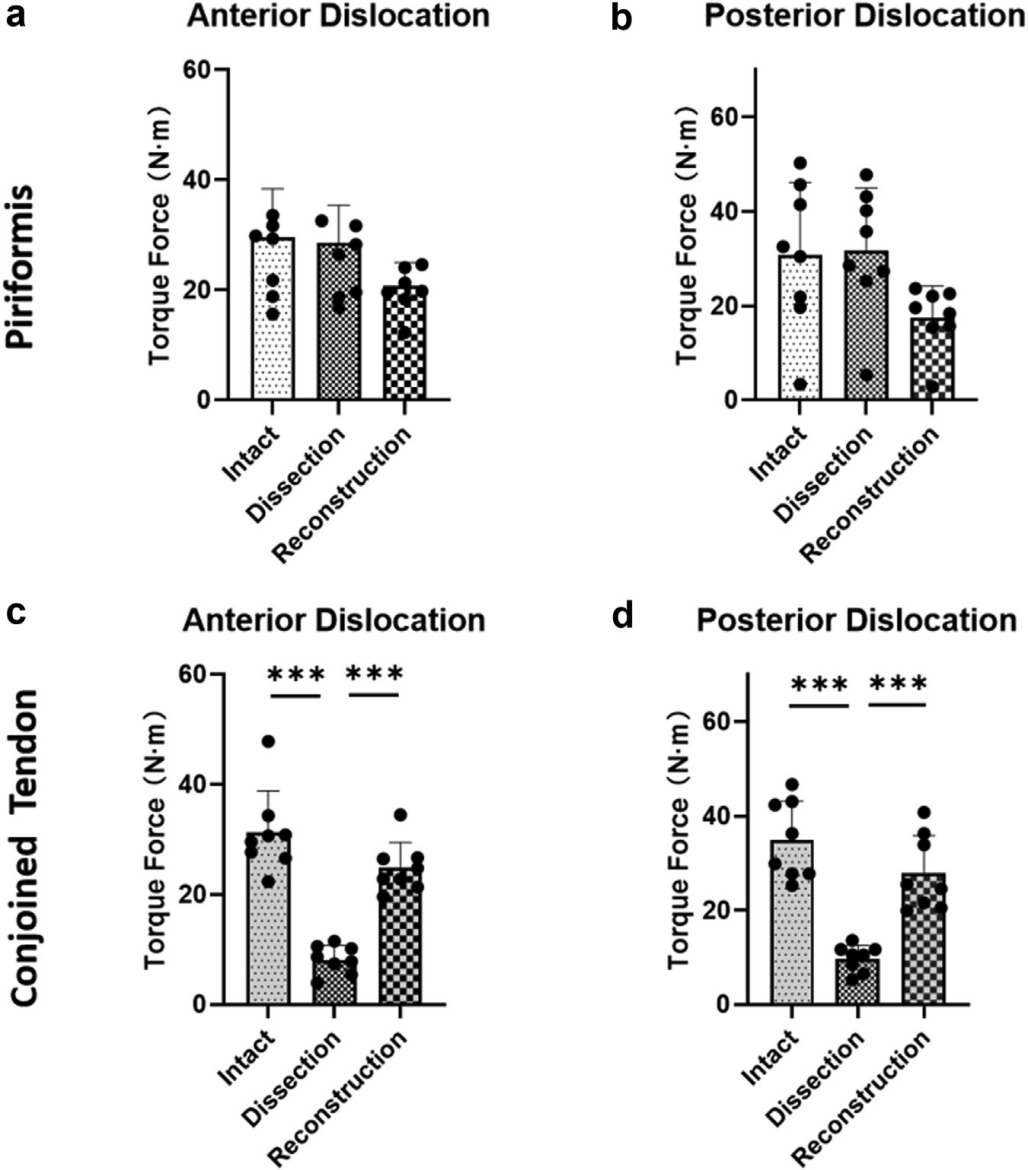
The torsional force required for simulating anterior and posterior dislocations with intact PT and CT tendons, as well as the torsional force required after severing and reconstructing these tendons. The black dots in the bar graphs represent samples. "∗": .01 < *P* < .05; "∗∗": .001 < *P* < .01; "∗∗∗": *P* < .001.

In the PT group, our findings revealed the following: the mean torque required for anterior dislocation of the hip with the tendon intact was 28.84 ± 9.31 N.m. After the removal of the PT, the torque required for anterior dislocation was 22.24 ± 4. 53 N.m. Notably, there was no statistically significant difference between the 2 measurements (*P* = .7741). For posterior dislocation, the mean torque before and after PT removal was 30.7 ± 15.5 N.m; 31.7 ± 13.4 N.m, again with no significant difference observed (*P* = .9854). Furthermore, following the reconstruction of the piriformis muscle, the torque required for both anterior and posterior dislocation was 20.04 ± 2.67 N.m and 17.5 ± 6.7 N.m, respectively, with no significant increase in the required torque observed (*P* = .3504; *P* = .0801). (Fig. 2a and b)

In the CT group, a substantial reduction in the torque required for anterior dislocation of the hip was observed following the resection of the CT, with values dropping from 31.2 ± 7.6 N.m to 8.18 ± 2.6 N.m, (*P* < .001). Similarly, posterior dislocation required less torque after CT resection, decreasing from 34.9 ± 8.3 N.m to 9.8 ± 2.8 N.m, (*P* < .001). However, the torque required for both anterior and posterior dislocation significantly increased after the reconstruction of the CT, with values reaching 24.9 ± 4.6 N.m and 31.7 ± 13.4 N.m, respectively (both *P* < .001) (Table 2, Fig. 2c and d).

**Table 2:** Torque during anterior and posterior dislocation in PT and CT groups.

Groups	Anterior dislocation			Posterior dislocation		
	Torque (N.m)	SD	*P*	Torque (N.m)	SD	*P*
PT						
Intact	28.84	9.31	.774	30.7	15.5	.985
Dissection	22.24	4.53		31.7	13.4	
Reconstruction	20.04	2.67	.350	17.5	6.7	.080
CT						
Intact	31.2	7.6	<.001∗∗∗	34.9	8.3	<.001∗∗∗
Dissection	8.18	2.6		9.8	2.8	
Reconstruction	24.9	4.6	<.001∗∗∗	31.7	13.4	<.001∗∗∗

PT, piriformis tendon; CT, cojoined tendon; SD, standard deviation.

*P*-values determined using one-way analysis of variance. "∗∗∗": *P* < .001.

## Discussion

This study investigates the roles of the PT and the CT in hip stability post-total hip arthroplasty. We measured the torque required to dislocate the hip in cadavers, comparing it with intact group, PT group, and CT group. Our results indicate that the CT plays a more significant role than the PT in maintaining hip stability and preventing dislocation. This finding underscores the importance of understanding the roles of tendons in surgical planning for hip arthroplasty.

Hip dislocation occurs when the femoral head's center of rotation is misaligned with the acetabulum's center, often indicated by the greater trochanter's position [8,20,34,35]. Some research suggests that the PT contributes to maintaining hip stability during such dislocations. [21,25,26,36]. However, the CT, which comprises the superior gemellus, inferior gemellus, and obturator internus, is more influential. Comparing to the pear-shaped, flatter piriformis muscle, the CT’s larger size might enhance its role in stabilizing the hip. Notably, the piriformis originates from the second to fourth sacral vertebrae and inserts into the greater trochanter, whereas the CT originates from the foramen ovale and pubis, ending near the piriformis' stopping point on the greater trochanter [24,37]. The CT’s origin and endpoints are more closely aligned, which seems to restrict the internal rotation of the greater trochanter during posterior dislocations. Conversely, in cases of anterior dislocation, the hip joint undergoes external rotation accompanied by posterior extension. During such events, the piriformis muscle becomes relatively shorter and assumes a relaxed state, reducing its effect on the movement and stability of the greater trochanter. [17].

Our study has several limitations. Despite numerous attempts to ensure standardized placement of the cups through intraoperative fluoroscopy, indirect measurements were not always consistent and did not accurately simulate hip dislocation every time. We adjusted the offset by varying the size of the stems and heads and indirectly measured the lengths of the lower limbs using fluoroscopy of osseous landmarks during surgery. However, these methods were not always standardized. Regarding the femoral stem, we had to rely on the operator's experience. Despite the operator's expertise ensuring a uniform placement with good compression fit and no rotation of the prosthesis in the medullary cavity, variability remains. Additionally, our study did not include the obturator externus tendon, another crucial structure for hip stability, which can act as a lever during dislocations, affecting the motion of the greater trochanter and overall hip stability [17,38,39,40,41]. Furthermore, the joint capsule, particularly the iliofemoral, pubic, and sciatic ligaments, also play significant roles in stabilizing the hip and might exhibit antagonistic or synergistic effects on the CT. Other factors such as age, gender, body weight, and adductor strength also influence hip stability [7,11,42]. Moreover, this study was conducted using cadaveric specimens, which exclude active muscle contraction. Muscle activity tended to be more complex in the human body, along with various types of tissue healing and scar repair. These factors also had an impact on hip stability. Postoperative dislocations can be affected by bone structure, muscle and soft tissue integrity, and neuropathic conditions. While this study concentrated on specific anatomical aspects, future research should encompass a broader and more detailed investigation into these various contributing factors [5,9,36,34,43].

## Conclusions

Hip dislocation, while conceptually straightforward, is a multifaceted phenomenon influenced by an array of factors, among which soft tissue structures constitute a significant component. Within this intricate framework, we think the comparatively marginal role of the PT in sustaining hip stability. Conversely, the CT appears to assume a more critical, perhaps even pivotal, role in this regard.

As a result, maintaining the integrity of the CT should be a concern in THA.

## Acknowledgments

The authors would like to thank Professor Chunfeng Zhao’s edition and revision, who is the consultant of Department of Orthopedic Surgery of Mayo Clinic.

## Funding

Gongyin Zhao has received the support from Technology Development Foundation of the 10.13039/501100007289Nanjing Medical University (grant number: NMUB20210050). Yuji Wang has received the support from Clinical Research Project of Changzhou Medical Center of Nanjing Medical University (grant number: CMCC2022XX).

## Conflicts of interest

The authors declare there are no conflicts of interest.

For full disclosure statements refer to https://doi.org/10.1016/j.artd.2024.101487.

## CRediT authorship contribution statement

**Gongyin Zhao:** Writing – review & editing, Writing – original draft, Project administration, Investigation, Funding acquisition, Formal analysis, Data curation, Conceptualization. **Chenyu Zhao:** Methodology, Investigation. **Hongwei Bao:** Methodology, Investigation. **Junting Liu:** Methodology, Investigation, Data curation. **Baojun Zhou:** Methodology, Investigation, Data curation. **Yuji Wang:** Writing – review & editing, Writing – original draft, Supervision, Investigation, Funding acquisition, Formal analysis, Data curation.

## Appendix A Supplementary Data

Conflict of Interest Statement for All the Authors
