# Supplementary material for: Role of the Conjoined Tendon in Hip Stability Post-Total Hip Arthroplasty: Insights From a Direct Anterior Approach Cadaver Study
Source: Arthroplast Today. 2024 Oct 15;30:101487. doi: 10.1016/j.artd.2024.101487 (PMC11530807; doi:10.1016/j.artd.2024.101487)
Supplement: Conflict of Interest Statement for All the Authors [file mmc1.pdf]

INDIVIDUAL CONFLICT OF INTEREST STATEMENT

American Association of Hip and Knee Surgeons

(Adopted from the American Academy of Orthopaedic Surgeons disclosure statement)

The following form **must be filled out completely and submitted by each author (example, 6 authors, 6 forms). All items require a response. If there is no relevant disclosure for a given item, enter "*None.*"**

Manuscript Title: Role of the Conjoined Tendon in Hip Stability Post-Total Hip Arthroplasty: Insights from a Direct Anterior Approach Cadaver Study

---

1. Royalties from a company or supplier (The following conflicts were disclosed)

   No.

2. Speakers bureau/paid presentations for a company or supplier (The following conflicts were disclosed)

   No.

3A. Paid employee for a company or supplier (The following conflicts were disclosed)

   No.

3B. Paid consultant for a company or supplier (The following conflicts were disclosed)

   No.

3C. Unpaid consultants for a company or supplier (The following conflicts were disclosed)

   No

4. Stock or stock options in a company or supplier (The following conflicts were disclosed)

   No.

5. Research support from a company or supplier as a Principal Investigator (The following conflicts were disclosed)

   No.

6. Other financial or material support from a company or supplier (The following conflicts were disclosed)

   No.

7. Royalties, financial or material support from publishers (The following conflicts were disclosed)

   No.

8. Medical/Orthopaedic publications editorial/governing board (The following conflicts were disclosed) No.

9. Board member/committee appointments for a society (The following conflicts were disclosed) No.

Each author must sign AND print or type his/her name, date and submit a separate form

In addition, one BLINDED Conflict of Interest form (no author names used) should be submitted per manuscript with all author disclosures.

Author Name (Print or Type) Hongwei Bao Author Signature Hongwei Bao Date 12/01/2023

INDIVIDUAL CONFLICT OF INTEREST STATEMENT

American Association of Hip and Knee Surgeons

(Adopted from the American Academy of Orthopaedic Surgeons disclosure statement)

The following form **must be filled out completely and submitted by each author (example, 6 authors, 6 forms). All items require a response. If there is no relevant disclosure for a given item, enter "*None.*"**

Manuscript Title: Role of the Conjoined Tendon in Hip Stability Post-Total Hip Arthroplasty: Insights from a Direct Anterior Approach Cadaver Study

1. Royalties from a company or supplier (The following conflicts were disclosed)

   No.

2. Speakers bureau/paid presentations for a company or supplier (The following conflicts were disclosed)

   No.

3A. Paid employee for a company or supplier (The following conflicts were disclosed)

   No.

3B. Paid consultant for a company or supplier (The following conflicts were disclosed)

   No.

3C. Unpaid consultants for a company or supplier (The following conflicts were disclosed)

   No

4. Stock or stock options in a company or supplier (The following conflicts were disclosed)

   No.

5. Research support from a company or supplier as a Principal Investigator (The following conflicts were disclosed)

   No.

6. Other financial or material support from a company or supplier (The following conflicts were disclosed)

   No.

7. Royalties, financial or material support from publishers (The following conflicts were disclosed)

   No.

8. Medical/Orthopaedic publications editorial/governing board (The following conflicts were disclosed) No.

9. Board member/committee appointments for a society (The following conflicts were disclosed) No.

Each author must sign AND print or type his/her name, date and submit a separate form

In addition, one BLINDED Conflict of Interest form (no author names used) should be submitted per manuscript with all author disclosures.

Author Name (Print or Type) Gongyin Zhao Author Signature Gongyin Zhao Date 12/01/2023

INDIVIDUAL CONFLICT OF INTEREST STATEMENT

American Association of Hip and Knee Surgeons

(Adopted from the American Academy of Orthopaedic Surgeons disclosure statement)

The following form **must be filled out completely and submitted by each author (example, 6 authors, 6 forms). All items require a response. If there is no relevant disclosure for a given item, enter "*None.*"**

Manuscript Title: Role of the Conjoined Tendon in Hip Stability Post-Total Hip Arthroplasty: Insights from a Direct Anterior Approach Cadaver Study

---

1. Royalties from a company or supplier (The following conflicts were disclosed)

No.

2. Speakers bureau/paid presentations for a company or supplier (The following conflicts were disclosed)

No.

3A. Paid employee for a company or supplier (The following conflicts were disclosed)

No.

3B. Paid consultant for a company or supplier (The following conflicts were disclosed)

No.

3C. Unpaid consultants for a company or supplier (The following conflicts were disclosed)

No

4. Stock or stock options in a company or supplier (The following conflicts were disclosed)

No.

5. Research support from a company or supplier as a Principal Investigator (The following conflicts were disclosed)

No.

6. Other financial or material support from a company or supplier (The following conflicts were disclosed)

No.

7. Royalties, financial or material support from publishers (The following conflicts were disclosed)

No.

8. Medical/Orthopaedic publications editorial/governing board (The following conflicts were disclosed) No.

9. Board member/committee appointments for a society (The following conflicts were disclosed) No.

Each author must sign AND print or type his/her name, date and submit a separate form

In addition, one BLINDED Conflict of Interest form (no author names used) should be submitted per manuscript with all author disclosures.

Author Name (Print or Type) Yuji Wang Author Signature Yuji Wang Date 12/01/2023

INDIVIDUAL CONFLICT OF INTEREST STATEMENT

American Association of Hip and Knee Surgeons

(Adopted from the American Academy of Orthopaedic Surgeons disclosure statement)

The following form **must be filled out completely and submitted by each author (example, 6 authors, 6 forms). All items require a response. If there is no relevant disclosure for a given item, enter "*None.*"**

Manuscript Title: Role of the Conjoined Tendon in Hip Stability Post-Total Hip Arthroplasty: Insights from a Direct Anterior Approach Cadaver Study

---

1. Royalties from a company or supplier (The following conflicts were disclosed)

No.

2. Speakers bureau/paid presentations for a company or supplier (The following conflicts were disclosed)

No.

3A. Paid employee for a company or supplier (The following conflicts were disclosed)

No.

3B. Paid consultant for a company or supplier (The following conflicts were disclosed)

No.

3C. Unpaid consultants for a company or supplier (The following conflicts were disclosed)

No

4. Stock or stock options in a company or supplier (The following conflicts were disclosed)

No.

5. Research support from a company or supplier as a Principal Investigator (The following conflicts were disclosed)

No.

6. Other financial or material support from a company or supplier (The following conflicts were disclosed)

No.

7. Royalties, financial or material support from publishers (The following conflicts were disclosed)

No.

8. Medical/Orthopaedic publications editorial/governing board (The following conflicts were disclosed) No.

9. Board member/committee appointments for a society (The following conflicts were disclosed) No.

Each author must sign AND print or type his/her name, date and submit a separate form

In addition, one BLINDED Conflict of Interest form (no author names used) should be submitted per manuscript with all author disclosures.

Author Name (Print or Type) Chenyu Zhao Author Signature Chenyu Zhao Date 12/01/2023

INDIVIDUAL CONFLICT OF INTEREST STATEMENT

American Association of Hip and Knee Surgeons

(Adopted from the American Academy of Orthopaedic Surgeons disclosure statement)

The following form **must be filled out completely and submitted by each author (example, 6 authors, 6 forms). All items require a response. If there is no relevant disclosure for a given item, enter "*None.*"**

Manuscript Title: Role of the Conjoined Tendon in Hip Stability Post-Total Hip Arthroplasty: Insights from a Direct Anterior Approach Cadaver Study

---

1. Royalties from a company or supplier (The following conflicts were disclosed)

No.

2. Speakers bureau/paid presentations for a company or supplier (The following conflicts were disclosed)

No.

3A. Paid employee for a company or supplier (The following conflicts were disclosed)

No.

3B. Paid consultant for a company or supplier (The following conflicts were disclosed)

No.

3C. Unpaid consultants for a company or supplier (The following conflicts were disclosed)

No

4. Stock or stock options in a company or supplier (The following conflicts were disclosed)

No.

5. Research support from a company or supplier as a Principal Investigator (The following conflicts were disclosed)

No.

6. Other financial or material support from a company or supplier (The following conflicts were disclosed)

No.

7. Royalties, financial or material support from publishers (The following conflicts were disclosed)

No.

8. Medical/Orthopaedic publications editorial/governing board (The following conflicts were disclosed) No.

9. Board member/committee appointments for a society (The following conflicts were disclosed) No.

Each author must sign AND print or type his/her name, date and submit a separate form

In addition, one BLINDED Conflict of Interest form (no author names used) should be submitted per manuscript with all author disclosures.

Author Name (Print or Type) Junting Liu Author Signature Junting Liu Date 12/01/2023

INDIVIDUAL CONFLICT OF INTEREST STATEMENT

American Association of Hip and Knee Surgeons

(Adopted from the American Academy of Orthopaedic Surgeons disclosure statement)

The following form **must be filled out completely and submitted by each author (example, 6 authors, 6 forms). All items require a response. If there is no relevant disclosure for a given item, enter "*None.*"**

Manuscript Title: Role of the Conjoined Tendon in Hip Stability Post-Total Hip Arthroplasty: Insights from a Direct Anterior Approach Cadaver Study

---

1. Royalties from a company or supplier (The following conflicts were disclosed)

No.

2. Speakers bureau/paid presentations for a company or supplier (The following conflicts were disclosed)

No.

3A. Paid employee for a company or supplier (The following conflicts were disclosed)

No.

3B. Paid consultant for a company or supplier (The following conflicts were disclosed)

No.

3C. Unpaid consultants for a company or supplier (The following conflicts were disclosed)

No

4. Stock or stock options in a company or supplier (The following conflicts were disclosed)

No.

5. Research support from a company or supplier as a Principal Investigator (The following conflicts were disclosed)

No.

6. Other financial or material support from a company or supplier (The following conflicts were disclosed)

No.

7. Royalties, financial or material support from publishers (The following conflicts were disclosed)

No.

8. Medical/Orthopaedic publications editorial/governing board (The following conflicts were disclosed) No.

9. Board member/committee appointments for a society (The following conflicts were disclosed) No.

Each author must sign AND print or type his/her name, date and submit a separate form

In addition, one BLINDED Conflict of Interest form (no author names used) should be submitted per manuscript with all author disclosures.

Author Name (Print or Type) Baojun Zhou Author Signature Baojun Zhou Date 12/01/2023

INDIVIDUAL CONFLICT OF INTEREST STATEMENT

American Association of Hip and Knee Surgeons

(Adopted from the American Academy of Orthopaedic Surgeons disclosure statement)

The following form **must be filled out completely and submitted by each author (example, 6 authors, 6 forms). All items require a response. If there is no relevant disclosure for a given item, enter "*None.*"**

Manuscript Title: Role of the Conjoined Tendon in Hip Stability Post-Total Hip Arthroplasty: Insights from a Direct Anterior Approach Cadaver Study

1. Royalties from a company or supplier (The following conflicts were disclosed)

No.

2. Speakers bureau/paid presentations for a company or supplier (The following conflicts were disclosed)

No.

3A. Paid employee for a company or supplier (The following conflicts were disclosed)

No.

3B. Paid consultant for a company or supplier (The following conflicts were disclosed)

No.

3C. Unpaid consultants for a company or supplier (The following conflicts were disclosed)

No

4. Stock or stock options in a company or supplier (The following conflicts were disclosed)

No.

5. Research support from a company or supplier as a Principal Investigator (The following conflicts were disclosed)

No.

6. Other financial or material support from a company or supplier (The following conflicts were disclosed)

No.

7. Royalties, financial or material support from publishers (The following conflicts were disclosed)

No.

8. Medical/Orthopaedic publications editorial/governing board (The following conflicts were disclosed) No.

9. Board member/committee appointments for a society (The following conflicts were disclosed) No.

Each author must sign AND print or type his/her name, date and submit a separate form

In addition, one BLINDED Conflict of Interest form (no author names used) should be submitted per manuscript with all author disclosures.

Author Name (Print or Type) Farbod Yousefi Author Signature Farbod Yousefi Date 12/01/2023
